# An Update in Computational Methods for Environmental Monitoring: Theoretical Evaluation of the Molecular and Electronic Structures of Natural Pigment–Metal Complexes

**DOI:** 10.3390/molecules29071680

**Published:** 2024-04-08

**Authors:** Gabriella Josephine Maranata, Sandra Megantara, Aliya Nur Hasanah

**Affiliations:** 1 Department of Pharmaceutical Analysis and Medicinal Chemistry, Faculty of Pharmacy, Universitas Padjadjaran, Jl. Raya Bandung Sumedang KM 21, 5, Jatinangor, Sumedang 45363, Indonesias.megantara@unpad.ac.id (S.M.); 2 Drug Development Study Centre, Faculty of Pharmacy, Universitas Padjadjaran, Sumedang 45363, Indonesia

**Keywords:** metals, natural pigments, computational methods, quantum mechanical methods, molecular dynamics

## Abstract

Metals are beneficial to life, but the presence of these elements in excessive amounts can harm both organisms and the environment; therefore, detecting the presence of metals is essential. Currently, metal detection methods employ powerful instrumental techniques that require a lot of time and money. Hence, the development of efficient and effective metal indicators is essential. Several synthetic metal detectors have been made, but due to their risk of harm, the use of natural pigments is considered a potential alternative. Experiments are needed for their development, but they are expensive and time-consuming. This review explores various computational methods and approaches that can be used to investigate metal–pigment interactions because choosing the right methods and approaches will affect the reliability of the results. The results show that quantum mechanical methods (ab initio, density functional theory, and semiempirical approaches) and molecular dynamics simulations have been used. Among the available methods, the density functional theory approach with the B3LYP functional and the LANL2DZ ECP and basis set is the most promising combination due to its good accuracy and cost-effectiveness. Various experimental studies were also in good agreement with the results of computational methods. However, deeper analysis still needs to be carried out to find the best combination of functions and basis sets.

## 1. Introduction

Metals are relatively abundant and can be found in almost all aspects of life. They provide many benefits, including serving as enzyme cofactors, participation in regulatory systems, and the ability to support protein structures [1,2]. However, some metals can be very dangerous and cause serious problems, especially when present at high concentrations for a long period of time [3,4,5,6,7,8,9]. Based on the danger they pose, metals can be classified into two distinct groups, namely heavy metals and non-heavy metals.

Heavy metals have high densities and atomic weights and are toxic even in very small amounts [4]. These elements are naturally present within Earth’s crust [10,11] and released into the environment through various naturally occurring processes, anthropogenic activities, and bio-geo processes [12,13]. The term heavy metal usually refers to metals and metalloids that are related to toxicity and pollution [14,15]. Almost all heavy metals are environmental pollutants and have a markedly negative impact on biota. Today, the toxicity of heavy metals is becoming an increasingly rampant problem from ecological, evolutionary, health, and, especially, environmental perspectives [15,16,17,18,19]. Heavy metal contamination can disturb the balance of both soil and aquatic ecosystems, which can lead to a reduction in biota populations and even extinction [20,21]. Apart from the environment, heavy metals are dangerous for the body. For example, mercury (Hg) can disrupt the membrane potential and also disturb intracellular calcium (Ca) homeostasis [22], while cadmium (Cd) causes iron (Fe) deficiency by binding to aspartate, cysteine, glutamate, and histidine ligands [23]. Another example is arsenic (As), which affects the sulfhydryl groups in cells and thus disrupts cell respiration, mitosis, and enzyme function [9].

Non-heavy metals are generally not dangerous in small amounts, but if the amounts are excessive, they can also have negative impacts on the body. Fe, cobalt (Co), manganese (Mn), zinc (Zn), and copper (Cu) can actually be useful and are needed by our body, but toxic effects may occur when they are present in excess [11,24]. For example, Fe is a cofactor for various important proteins and enzymes but can also cause liver fibrosis and cirrhosis [25]; increase the potential for heart failure and arrhythmias [26,27]; and increase the possibility of osteoporosis, hypothyroidism, and hypoparathyroidism [28]. In addition, Cu and chromium (Cr), among other non-heavy metals, have a very small beneficial concentration range [4].

To protect the health of people, regulatory organisations have proposed maximum amounts of certain metals (Table 1) [7,24,29,30]. In reality, the dangers of metals are very difficult to control because often the amount of exposure is not clearly known. At present, environmental pollution by metals, especially heavy metals, is an unavoidable threat and challenge, especially with increasing industrial activity without proper waste processing [31,32,33,34,35] and an increase in traffic volume [36]. Pollution spreads through the air, water, animal products, agricultural products, and various other components into the surroundings [37,38,39,40].

Metals can enter plants, animals, and humans in various ways, such as through the respiratory tract, food and drink, and even skin contact. Due to their abundance and the fact that they cannot be seen with the naked eye, metals can enter tissues unnoticed over long periods of time and accumulate in various organs, including the brain, kidneys, liver, and heart. After accumulation, they can disrupt normal biological systems and functions [11,14,16,17,41].

Detecting the presence of metals is crucial to ensuring that organisms and the environment are protected from their negative effects. Many researchers detect metal levels by using atomic absorption spectroscopy [42,43], flame spectroscopy [44], X-ray fluorescence spectrometry [45], inductively coupled plasma mass spectroscopy [36], neutron activation analysis, and inductively coupled plasma-optical emission spectrometry [46,47], among others. These methods show good selectivity and sensitivity; however, they lack efficiency when viewed in terms of time, energy, and costs. Moreover, the availability of instruments is still limited [20,39]. Due to existing limitations, the development of portable detectors that are efficient in terms of time, cost, and energy could represent a solution. While many portable detectors have been developed, some of them are prepared using reagents and materials that may cause danger and produce contamination. Therefore, using natural pigments in the development of portable metal detectors is a potential solution to create detectors that are safe for the environment, simple, do not require chemical reagents, and are efficient in terms of energy and time [48].

**Figure 1:**
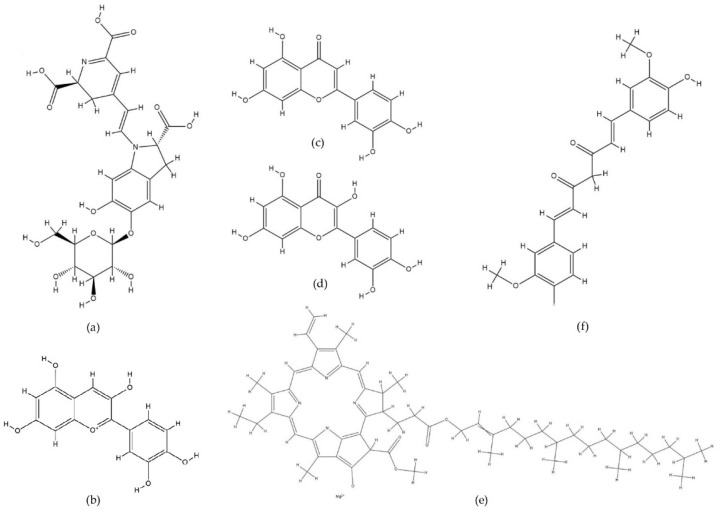
Chemical structure of natural pigments: (**a**) betanin, a derivative of the betalain pigment; (**b**) cyanidin; (**c**) luteolin; (**d**) quercetin; (**e**) chlorophyll; (**f**) curcumin.

Natural pigments—coloured compounds from various sources in nature—can be a solution to this need [49]. These pigments provide colour to fruits and vegetables—for example, betalains produce a reddish-yellow colour, anthocyanins give a reddish-blue colour, carotenoids give a yellow-to-orange colour, and chlorophylls give a green colour [50]. Pigments have chromophores in their structure, so they absorb light in the ultraviolet and visible regions [51]. The chromophore group captures energy by attracting electrons from lower to higher energy orbitals, and then the remaining energy that is not absorbed is reflected and appears as colour to the human eye [50]. Examples of natural pigments that are often found and used in the development of metal detectors from natural pigments can be seen in Figure 1.

Natural pigments can form complexes with metals, and these complexes result in colour changes. Hence, portable metal detectors could incorporate natural pigments to detect metals based on colour changes [52,53,54]. Several studies have shown that pigments and metals are capable of forming complexes. Wybraniec et al. (2013) [55] reported that betalains can interact with nickel (Ni) and Cu, decreasing the absorbance of the pigment. Saithongdee et al. (2014) [56] reported that curcumin forms a complex with Fe and provides a shift from yellow to brown. Khaodee et al. (2014) [57] reported that cyanidin forms colour complexes with Cu, lead (Pb), and aluminium (Al), showing a shift from purple to blue, as well as with Fe, which shows a shift from purplish pink to blue. Several other studies have also been conducted related to the development of natural pigments as metal detectors. In Table 2, data from some studies related to this topic are shown, along with the colour changes and detection limits that can be visually observed.

Computational methods can be used to efficiently study the interaction of metals with natural pigments. Direct experiments in the laboratory may take more time, cost more, and produce more waste. Computational studies are more efficient and able to determine the interactions that occur and other parameters [64,65]. Computational calculations will be very useful in predicting the possibility of colour shifts because changes in the ground-state geometry will affect the absorption peak shift [66]. A small change in the basic geometry of a molecule can trigger larger shape and chemical changes, including optical properties, distribution of electrons, and electronic structure, that shift the wavelengths of light it absorbs, which is directly related to the colour change that can be observed by the naked eye [39,67,68]. Until now, there has been no review that discusses this field, even though this preliminary research is very important and useful. Therefore, we provide an overview of the computational methods that can be utilised to study metal–natural pigment interactions. We also discuss the validation of these computational methods with experimental results.

## 2. Computational Methods for Studying Metal–Pigment Interactions in General

Computational methods have been used to study metal–natural pigment interactions for several years. During this time, they have improved and become increasingly well known. Studying the bonds and interactions between metals and pigments using computational methods is an important step before laboratory research; it can lead to more targeted studies that require fewer resources, costs, and energy. In addition, computational methods can overcome several limitations, and consistent, detailed experimental information at the structural and energetic levels can be obtained [69].

Due to its various advantages, computational study is now widely considered for use in various fields. Various calculations and predictions can be carried out using computational methods, for example in studying molecular system energy [70,71], gibbs free energy [64], binding energy [72], bond length and bond angle [73,74], natural bond orbitals [65], HOMO and LUMO [71,75,76], stability of the complexes [64,77], optical properties [75], and molecular dynamics studies [78]. Apart from that, this computational method can also be used to predict the electronic structure of a system and, thus, the distribution of electrons in the system [79,80].

In the development of natural pigments as metal detectors, one of the parameters that is directly related to metal detector development is excitation energy. Excitation energy is related to colour changes that may occur before and after the pigment interacts with the metal. The colour that an object possesses is determined by the wavelengths of light that are absorbed and reflected by that object, where the wavelength will depend on the structure of the molecules. When bonds are formed between natural pigments and metals, the electron configuration will undergo changes that also affect molecular orbits and energy levels. This is what will affect the occurrence of wavelength shifts and observed colour changes. In developing a metal detector from natural pigments, the excitation energy can be used to predict whether there is a change in colour and how the colour changes [71,81]. However, before studying the excitation energy of a molecule, studying the interactions that occur through analysing bond energy, total energy, affinity, and ground-state energy calculations is also important to do as a preliminary study to find out whether the bond will be formed between natural pigments and metals because, to produce colour changes, natural pigments must be able to bind to metal [17,82]. Apart from that, various other calculations such as the stability of the complexes, optical properties, and the geometry of the complexes formed can also be very helpful in the development of metal detectors from natural pigments [64,75,83].

Various computational methods and approaches are available to study the parameters mentioned above, and each method used and each approach applied will impact the results of the study carried out. Therefore, it is important to know what method is most appropriate and what kind of approach can be taken. It should be noted that the selection of an appropriate method will also be influenced by various factors and must be based on the specific needs and goals of the study, including what parameters are to be studied. In addition, it is also important to find an optimal combination of basis sets, methods, and approaches through multi-level approaches so that the results obtained have a balance between efficiency, accuracy, and robustness [84,85].

As mentioned earlier, there are many factors that can influence the outcome of computational calculations, which must also be taken into consideration. The main thing that must be considered is, of course, the molecule to be investigated [86], but apart from that, there are several other factors, such as the presence of other molecules in the system like co-pigments, pH [78,86], temperature [87,88], and also the solvent effect [89,90]. Solvents are capable of causing the occurrence of spectral shifts and, as explained earlier, the wavelength of the complex formed becomes a direct parameter that can be studied in the development of metal detectors from natural pigments. Solvents can cause changes in the ground and the excited state energy of molecules, so they can cause shifts in the absorption or emission spectrum of molecules. Thus, it can be concluded that the optimal wavelength in one solvent will be different than in another, as well as in the gas and solution phases [91]. The interactions between the molecules and the solvent can be studied using various methods. Explicit evaluation can be carried out using various methods, including free energy methods such as thermodynamic integration or metadynamics, where molecular dynamic-based sampling techniques and Monte Carlo methods can be carried out. This method can provide more detailed data compared to the implicit method, but the computational cost is relatively high [92]. On the other hand, implicit evaluation can also be carried out, where, in this model, the solvent is treated as a structureless continuum with certain dielectric and interface properties. In this approach, the contributions to the solvation-free energy of several models, such as conductor-like screening (COSMO) and polarizable continuum models (PCMs), can be applied [92,93]. Usually, method selection is based on a balance between calculation speed, cost, and expected accuracy. Data from various methods is also need to be taken into consideration in selecting the appropriate method [91]. These two approaches can also be combined to obtain more optimal results with better efficiency [94,95,96].

## 3. Quantum Mechanical Methods to Study Metal–Pigment Interactions

**Figure 2:**
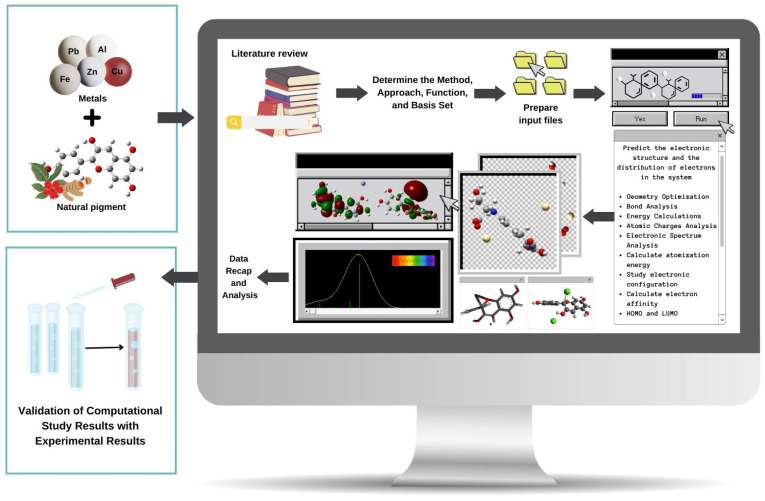
Illustration of the computational study flow to study the interaction between natural pigments and metals.

Quantum mechanical methods describe the electronic structure of a system to estimate the distribution of electrons in the system. These methods also allow one to evaluate the ability of pigments and metals to form complexes, including the possibility of bond formation, the structures formed, bond distances, bond energies, and the stability of the complex formed [80,97]. The major quantum mechanical approaches are the ab initio method, DFT, and semiempirical methods. Several studies have used these methods to study the bonds between metals and pigments. Below, we discuss the quantum mechanical methods in greater detail. The study flow of computational chemistry using quantum mechanical methods to study the interaction between natural pigments and metals can be seen in Figure 2.

### 3.1. Ab Initio Methods

Ab initio methods involve calculations that are derived directly from theoretical principles without requiring experimental data. There are several systematic approaches, including solving differential equations or Born–Oppenheimer approximation. One of the most widely used approaches to studying metal–pigment interactions is the HF calculation with the central field approximation method [98]. Table 3 presents studies that have used ab initio methods to study metal–pigment interactions.

Linnanto and Korppi (2004) [101] used the HF/6-31G* approach to calculate fully optimised structures and to calculate atomic charges of methyl bacteriochlorophyllides a, b, g, and h, which is a pigment responsible for photosynthesis in bacteria [105], and the magnesium (Mg)–bacteriochlorin complex. The authors also carried out optimisation using (DFT/B3LYP/6-31G*). In addition, the CIS/6-31G* and CIS/6-311G** configurations, as well as the HF/6-31G* and HF/6-311G** methods, have also been utilised to calculate the associated spectroscopic transition energy of the chromophores. The HF approach provides the structure in a vacuum; the four coordinated Mg atoms of bacteriochlorin are positioned centrally, nearly in the plane of the ring. The outcomes were consistent with calculations by other methods (e.g., DFT/B3LYP/6-31G*) and some of the earlier X-ray structure study results [106,107,108]. These results are consistent with semiempirical [101] and time-dependent DFT [109] calculations. The ab initio and DFT calculations produced slight differences in energy transitions compared with the experimental results. The bond length and coordinates have a strong influence on transition energy calculations, and because there are slight differences in the results from the methods used, the transition energy results are slightly different. The electronic transition calculations using ab initio methods are systematically higher than DFT calculations. Moreover, DFT with B3LYP and ZINDO/S transition energies produced the finest correlation coefficient between the calculated and experimentally determined transition energies [101].

Cao et al. (2014) [103] used the ab initio method with the HF/6-31G* level of theory and the Gaussian 09 program for full geometry optimisation and to determine whether there is an influence of additional functional groups in the induction of atomic charge distribution and the influence of methylation position on luteolin and quercetin. Quercetin and luteolin are flavonoids, which are pigments with a phenolic structure that can be found in vegetables, fruits, and medicinal plants [80,110]. Before carrying out calculations, the authors compared luteolin and quercetin regarding functional groups, the type of bond that occurs, and the possibility of electron movement. Quercetin has an additional 3-OH group in its structure, while luteolin does not; the three -OH groups will donate electrons in quercetin with a delocalised π bond. With these considerations, the authors carried out calculations on a single molecule to determine whether the additional -OH group in quercetin induces a different atomic charge distribution on the two oxygen atoms in the luteolin and quercetin catechol groups. It is important to perform this initial assessment before the calculations to choose the most appropriate approach and get more information from the calculations. Following the preliminary analysis, the authors calculated the partial charges of the meta-O and para-O structures in luteolin and quercetin and noted that the meta-O structure is more nucleophilic because both luteolin and quercetin have more negative charges in their meta-O structure than in their para-O structure. Furthermore, the equivalent hydrogen in meta-OH is more attracted to the oxygen atom than it is in para-OH, meaning that meta-OH would have a larger reaction capacity for methylation than the para-OH structure [103].

Apart from being used to study the bonds that occur between pigments and metals, ab initio methods can also be used to see the effect of hydration on metal–pigment interactions. Hydration can impact the physicochemical properties of metals, pigments, and metal–pigment complexes; the interactions that occur; and the reactivity and stability of various systems. In addition, water molecules can impact the compounds and the complexes formed in diverse ways [39,111].

Overall, ab initio methods can be used to study metal–pigment interactions, including to optimise complex structures, analyse the dynamic properties, solvation capabilities, and hydration behaviour of a compound, band structures, polarisabilities, and atomic charges, and calculate energies. The HF method is widely used along with the 6-31G, 6-31G*, 6-31G**, and 6-31G(d) basis sets, both single basis sets and combinations of several basis sets. Deeper development of the method with various combinations of other approaches could be carried out. However, ab initio methods are not the main choice to study metal–natural pigment interactions due to their relatively poor level of accuracy. Although ab initio methods have not been used to study metal–pigment interactions in depth, they have been used to study the interactions between heavy metals and other compounds [112,113,114]. Thus, the use of this method to study heavy metal–natural pigment interactions can still be developed to determine its uses, advantages, and disadvantages for studying metal–pigment complexes.

### 3.2. Density Functional Theory (DFT)

DFT has been used since 1990 to calculate the electronic structure of various molecules; it is considered a powerful method for simulating chemical systems [115,116]. In addition, it has a good level of efficiency and accuracy [115]. DFT can be used to calculate atomisation energy or binding energy [17,103,116], bond lengths and angles [73,115], electronic configuration [116], electron affinity [115], ionisation potential [65,115], heat of formation [115], several types of nonbonding interactions [115], and geometric optimisation of a complex, especially for a system involving transition metals, which needs to consider electron correlation. In this method, the system of electrons that undergo interactions is mapped specifically into an effective non-interacting system with a similar total density [116,117]. Table 4 presents studies that have used DFT to study metal–pigment interactions.

DFT calculations are the most widely used method, including to study metal–pigment interactions [65,124,126,127,128,129,130,131]. This approach continues to be developed functionally, and to date, there are many methods with various applications that can be used. Sousa et al. (2007) [115] collected data related to the various DFT functionals available. They found that B3LYP was the most widely used DFT functional, with a frequency of 80% from 1990 to 2006. BLYP, B3PW9, BP86, and M05-2X were also relatively widely used functionals (3% each). B3LYP is widely used because it has several advantages: it is considered an accurate method with wide coverage and relatively affordable calculation costs. However, it has several shortcomings, such as the possibility of a decrease in the level of accuracy as the size of the system being investigated increases, as well as an underestimation of weak interactions [132]. This method can still be developed further to achieve higher accuracy and wider use because each available functional is very flexible, making it possible to combine one or more exchange functionals and correlation functionals [115,129,130,131,133].

DFT has been widely applied to study interactions between natural pigments and non-heavy metals (such as Al, Co, Cr, Cu, Fe, Mg, Mn, Ni, palladium [Pd], and Zn) and heavy metals (such as As, Cd, Hg, Pb, and tin [Sn]). Sun et al. (2008) [72] studied the chelation process between quercetin and Cr using DFT with the B3LYP functional combined with the LANL2DZ pseudopotential and the 6-31+G (d) basis set to determine the appropriate chelation site and to produce a stable metal–pigment complex. They calculated the bond energy and total energy and performed natural bond orbital analysis to analyse the charge transfer between pigment and metal. They found that Cr(III) tends to form a complex with quercetin at the 5-hydroxy-4-keto site. In addition, deprotonated quercetin attracts Cr better than its natural form. The authors also compared the computational results with direct synthesis and analysis of the bond characteristics using infrared spectroscopy and ultraviolet–visible (UV–Vis) spectroscopy. The experimental and computational experiments led to the same conclusions. Of the three accessible chelating sites, Cr(III) ions tended to chelate at the 5-hydroxy-4-keto site, where deprotonated quercetin has a higher chelating capacity than natural quercetin. The UV–Vis spectrum showed that deprotonated 5-OH coordinates the Cr(III) ion to form a complex. This study showed that a computational method provides accurate initial testing that is less expensive and relatively more efficient than direct experiments [72].

Guo et al. (2008) [117] studied the interaction between the butein anion and Mg, Cr, Fe, and Cu cations. Butein is a chalcone compound that can be found in various plants, such as dahlia, coreopsis, and lacquer tree [134]. Gou et al. (2008) used the B3LYP functional combined with the LANL2DZ pseudopotential and the 6-31+G (d) basis set, similarly to the approach employed by Sun et al. (2008) [72]. They performed metal–butein structure optimisation without any symmetry constraints, followed by a single-point calculation utilising a different basis set (6-311++G(d,p)). This method produced binding energies and total energy at the B3LYP/6-311++G(d,p) level to establish the most appropriate coordination and binding sites for a stable complex. Apart from that, the authors calculated natural bond orbitals with the same functional (B3LYP) but with a different basis set (6-311+G(d,p)) to study the charge transfer between butein and the metal cation. They found that the oxygen atom in positions 2′ and 9 was the most preferred. The calculations revealed that the strength of metal against metal is Mg^2+^ < Fe^2+^ < Cr^2+^ < Cu^2+^ in both the gas and liquid phases [117].

Leopoldini et al. (2006) [76] use the B3LYP/6-31G*/LANL2DZ level of DFT for geometric and electronic optimisation, followed by natural bond orbital analysis for better characterisation of Fe(II)–quercetin complexes. They performed single-point calculations with the 6-311++G** basis set for the nonmetal atoms to refine the electronic energies. Both neutral and deprotonated quercetin can form stable complexes with Fe, with an optimal binding energy at a metal-to-ligand ratio of 1:2. The most favoured bond positions are the oxygen atoms on carbons 3 and 4, and 5 and 4 with high binding energy values, which indicate that quercetin and Fe(II) are capable of forming strong complexes. In another study, the authors compared the calculations with experimental results based on a UV–Vis spectrum obtained computationally (B3LYP/6-31G*) with experimental results in methanol [135] and dimethylsulfoxide [136]. The authors identified absorption bands in the 370–390 nm region.

Eno et al. (2023) [65] studied the interaction between several metal ions (sodium [Na^+^], potassium [K^+^], Mg^2+^, Ca^2+^, and Al^3+^) with quercetin at the B3LYP/6-31+G(d) level of DFT. They examined reactivity, stability, origin of interaction, and the use of metal–quercetin complexes as potential antioxidants. The metal–pigment interactions were purely electrostatic. The K^+^–quercetin complex was the most stable, and the Ca^2+^–quercetin complex had the lowest values in the neutral form and was the most reactive. Based on its reactivity as an electron donor, in neutral conditions, the K^+^–quercetin complex is the most reactive. It has the lowest ionisation potential (382.78 kcal/mol). The Ca^2+^–quercetin and Mg^2+^–quercetin complexes have the lowest reactivity as electron donors, with an ionisation potential of 395.33 kcal/mol. In the deprotonated condition, the Mg^2+^–quercetin and Ca^2+^–quercetin complexes have a lower ionisation potential (363.95 kcal/mol), which indicates that in the deprotonated condition, the Ca^2+^–quercetin and Mg^2+^–quercetin complexes have a highest electron transfer reactivity [65].

Several studies have also used the M05-2X functional, which can be combined with several different basis sets to study the interaction between natural pigments and non-heavy metals. Malacaria et al. (2022) [64] analysed the complex between luteolin and Al, Fe, and Cu. They evaluated the properties and optimised geometry with the M05-2X hybrid function and the 6-31+G(d) basis set for carbon (C), hydrogen (H), oxygen (O), and Al atoms, and the relativistic compact Stuttgart/Dresden effective core potential with its related split valence for Cu and Fe atoms. They performed optimisation in a solvent by applying the solvent-based density model with a dielectric constant of 78.0. The authors calculated the free energy from the complexation reaction of the substitution of water molecules in the hexaaqua complex to compare the bonding abilities of various metals with pigments. Then, they refined the energy by single-point calculations using the same functional but with a larger basis set (6-311++G(d,p)). The computational approach shows that the Cu ion does not prefer specific sites in the pigment, while the Al and Fe atoms appear to prefer to bind to the 4-carbonyl-5-hydroxyl site on the A and C rings of the ligand [64]. Corrente et al. (2021) [17] also conducted the same research while comparing the results obtained with experimental experiments with the aid of ^1^HNMR (Proton Nuclear Magnetic Resonance) and ^13^CNMR (Carbon-13 Nuclear Magnetic Resonance) spectroscopy obtained during the titration experiment. The computational and experimental analysis provided consistent results. Quercetin tends to bind three cations at different coordination sites. Fe and Al are known to have no tendency to bind to certain sites from the three complexation sites on quercetin, whereas for Cu, sites 4–5 can be excluded [17].

Tanui et al. (2022) [123] used DFT with another approach to study the complex formed between morin, which is a yellowish pigment obtained from *Chlorophora tinctoria* wood [137], or quercetin, and Fe and Cu ions. They used the M06-2X/def2-SVP level of DFT to evaluate the stability of the complex formed. They found that morin has better stability than quercetin. It is also known that six-membered structures for morin and quercetin complexes with metals have slightly better stability compared with their respective five-membered counterparts. The two pigment complexes with Fe showed better stability compared with the complexes with Cu (based on a much lower energy) [123]. The M06-2X/def2-SVP functional can also be used to investigate the effect of the solvent by examining possible interactions between the solvent and the compound. Mollaamin et al. (2020) [138] investigated van der Waals density functions to examine the influence of solvents on delphinidin complexes with several non-heavy metals (Al, gallium (Ga), Cr, Fe, and Mg). They carried out accurate calculations by using m062x, m06-L, and m06-HF. These three methods have appropriate correspondence for non-bonded calculations between the compounds and the solvents used [138].

DFT can also be used to study heavy metal–natural pigment interactions (Table 5). Cornard et al. (2005) [135] studied the interaction between Pb(II) ions and quercetin in methanol, with the B3LYP/6-31G(d,p) level and LANL2DZ for Pb to optimise the geometry of the complex formed. Based on this research, three potential locations are associated with the possibility of bond formation. The highest complexation strength for the Pb(II) ion is in the catechol functional group, where, after complexation, the Pb atom is coordinated to the catechol group, which is on the same plane as quercetin, and the ligand stays completely planar. These results are consistent with the experimental electronic spectrum and complex vibrations. Based on the theoretical calculation results, there is a bathochromic shift of the wavelength band in the UV–Vis spectrum when complexation occurs. This is thought to occur due to the transfer of the ligand charge to the metal. By comparing the experimental and theoretical results, it is also possible to compare the chelating strength of Pb ions from three potential sites in quercetin, and the results show agreement that the catechol group has the best complex formation strength [135].

Pratiwi et al. (2020) [139] also used the B3LYP level and the 6-31G and LANL2DZ basis sets to study interactions and complex formation and to calculate the binding constants of three cationic porphyrin compounds, namely meso-tetrakis (N-methylpyridinium-4-yl) porphyrin (TMPyP), meso-tetrakis (1,3-dimethylimidazolium-2-yl) porphyrin (TDMImP), and meso-tetrakis (1,2-dimethylpyrazolium-4-yl) porphyrin (TDMPzP) with five heavy metals (Pb, Cd, Hg, Sn, and As). The authors discovered that the complexes between porphyrin cations and the five heavy metals occur spontaneously, as indicated by the lower energy compared with the free base form. The metal–TDMPzP complexes are less electrophilic and have greater chemical hardness compared with the metal–TMPyP or metal–TDMImP complexes. Hence, the metal–TDMPzP complexes are most stable, especially the complex with Cd, which has a lower energy level and electronic chemical potential, lower electrophilicity, and a higher binding constant as well as chemical hardness. On the other hand, TMPyP has a higher energy compared with the other two porphyrin cations, which shows that this compound has the lowest stability. The stability of the heavy metal–porphyrin complexes is also influenced by the ionic radii of the heavy metals, where it is known that Cd with a radius of 95 and Sn with a radius of 69 can enter the core of the porphyrin and form a complex, while Pb and Hg, which have larger radii (118 and 102, respectively), are unable to enter the porphyrin core because they are too large, thus forming the metalloporphyrin located above. On the other hand, As has a smaller radius than all other metals but tends to move out of the porphyrin plane, a phenomenon that is estimated to occur because the valence electrons of As ions are not suitable for interacting with the nitrogen in the porphyrin core. This result is also in line with experimental results, where it is known that TDMPzP will form a stabler metalloporphyrin complex when reacting with Cd^2+^ ions, with a binding constant of 5.1 × 10^7^ M^−1^ [139].

Overall, DFT methods can be used to study metal–natural pigment interactions with a fairly good level of accuracy. Among the many approaches that can be used, the B3LYP level has been the main choice to study natural pigment interactions with non-heavy metals and heavy metals. This is thought to be related to its good accuracy and wide coverage at an inexpensive cost. The LANL2DZ ECP and basis set are also often chosen and combined with other basis sets. A combination of various basis sets is often used to obtain better results and to complement the shortcomings of each basis set. Future development and research related to combinations in computational systems using DFT methods are very promising.

### 3.3. Semiempirical Methods to Study Metal–Pigment Interactions

Quantum mechanical calculations using semiempirical methods are generally similar to ab initio methods. These two methods can complement each other and overcome each other’s shortcomings. Compared with the ab initio approach, semiempirical calculations are relatively faster, but the results will depend greatly on the availability of parameters that correspond to the molecules analysed in the database. If the molecules are significantly different from the molecules in the database, then the calculations may deviate from the actual results. When carrying out inorganic analysis, semiempirical methods are used relatively rarely because the potential for errors that occur in the analysis tends to be higher, especially when transition elements are involved. Therefore, it is a good idea to compare semiempirical calculations with calculations using other methods or with experimental results [98,141]. Table 6 presents research that has used semiempirical methods to assess metal–pigment interactions.

Linnanto and Korppi (2004) [101] used a semiempirical method—at the ZINDO/S CIS (40,40) or (45,45) level in the ArgusLab (version 2.0.0) software—to calculate the transition energies and oscillation strengths of the Mg–bacteriochlorin complex and methyl bacteriochlorophyllides a, b, g, and h. They also combined the ZINDO/S CIS (40,40) level with the simple self-consistent reaction field (SCRF) method. The authors used a semiempirical method to assist in the estimation of the corresponding spectroscopic transition energy values of the chromophore and also studied the existence of dark electronic states in the system. Based on this research, pigment complexation will induce a dark electronic state below the main Soret transition, where this condition may have an important role in the energy transfer process [101].

Linnanto and Korppi (2004) [141] also used the semiempirical PM5 method to study orbitals on chlorophylls and bacteriochlorophylls. They optimised the structure of Mg–bacteriochlorin; Mg–chlorin; Mg–porphin; mesochlorophyll a; chlorophylls a, b, c1, c2, c3, and d; and bacteriochlorophylls a, b, c, d, e, f, g, and h, all with homologous structures. To ensure that the results were truly accurate, the authors compared the semiempirical PM5 method with several other quantum mechanical methods. The experimental, ab initio, and density functional results were suitable. Based on calculations and an experimental study with X-rays, all pigments are predicted to have a planar structure of porphyrin with four Mg atoms coordinated in a position almost in the centre of the porphyrin ring plane. More clearly, Mg is in the centre for Mg–bacteriochlorin, Mg–chlorin, and Mg–porphin and is slightly shifted out of the porphyrin plane for the chlorophylls and bacteriochlorophylls. The authors also used the PM5 method to investigate minimum energy and atomic charges. They found only a slight difference between the minimum energy obtained with PM5 and other methods in the form of B3LYP/6-31G* or HF/6-31G*. These results indicate that the efficiency of PM5 is quite good and computationally efficient, considering that the HF and DFT methods are more intensive [141].

Cornard and Merlin (2002) [74] conducted a semiempirical study using the AM1 Hamiltonian in the Hyperchem (version 5.0) program to evaluate structural modifications caused by the ligand via chelation of one and then two Al ions. Previously, quercetin’s shape and the complex it forms with Al had also been optimised by using this similar methodology. In an isolated state, the molecule adopts a staggered conformation with a O (O1–C2–C1′–C6′) angle of 26.7° [74,148]. Cornard and Merlin (2002) used the same semiempirical method to determine the geometric parameters of quercetin and two complexes that are generated after energy minimisation with the AM1 method and several stoichiometric ratios in different solvents (pure methanol, methanol + AcO^−^, and methanol + water + HCl). The authors also generated experimental electronic and vibrational spectra and compared these results with the calculations. They found that the 3-hydroxychromone group is the initial site implicated in the complex formation process in acidic media and pure methanol, whereas the catechol group has maximum chelating power in alkaline media. It is known that for large amounts of Al(III), with an Al(III)-to-quercetin ratio greater than 0.5, the same complex [Al_2_QR_4_]^3+^ is formed in methanol and alkaline media. However, in acidic media, the ortho-dihydroxyl group is never involved in complexation with Al(III). The authors also carried out a spectroscopic study to validate the calculations. The UV–Vis spectrophotometry results showed wavelength conformity with the calculation results. They also validated the calculations with Raman spectroscopy. The complex exhibits important spectral changes, especially in the range of 1500–1700 cm^−1^, and the mass formed by the two broad bands at 1612 and 1576 cm^−1^ in the quercetin spectrum is profoundly affected, indicating that the structure of both rings is modified by chelation [74].

These studies have shown that semiempirical methods can be used to study metal–natural pigment complexes, including how the complex forms, modifications that occur due to the chelation process, intermolecular behaviour, oscillation strengths, and corresponding spectroscopic transition energy values. In addition, semiempirical methods can be used to optimise structures and solvation. The calculations are consistent with several experimental results, but in terms of accuracy, the ab initio and DFT methods are still superior. Semiempirical methods have not yet been used to study heavy metal–natural pigment interactions in greater depth, even though they have been used to study interactions between heavy metals and other components [149,150].

For quantum mechanics, DFT methods are selected most often to study metal–pigment interactions. They have several advantages over ab initio and semiempirical methods, including a good level of efficiency and accuracy with a high level of implementation flexibility [115,129]. However, the suitability of DFT can also differ depending on the expected objectives. Linnanto and Korppi (2004) compared ab initio, DFT, and semiempirical methods to predict the structure of Mg–bacteriochlorin complexes. They found that the calculations using DFT with B3LYP/6-31G* had the smallest difference in transition energy compared with the experimental results. However, this study also showed that the size of the model system chosen and the environmental conditions for calculations greatly influence the reliability of the results obtained. Indeed, the pigment environment itself can alter the geometry of the chromophore and the electron density properties, which will change the energy level position. Therefore, it can be concluded that, apart from choosing the method that best suits the expected goals, it is crucial to choose the appropriate calculation environment conditions and system size [101].

## 4. Molecular Dynamics Methods to Study Metal–Pigment Interactions

**Figure 3:**
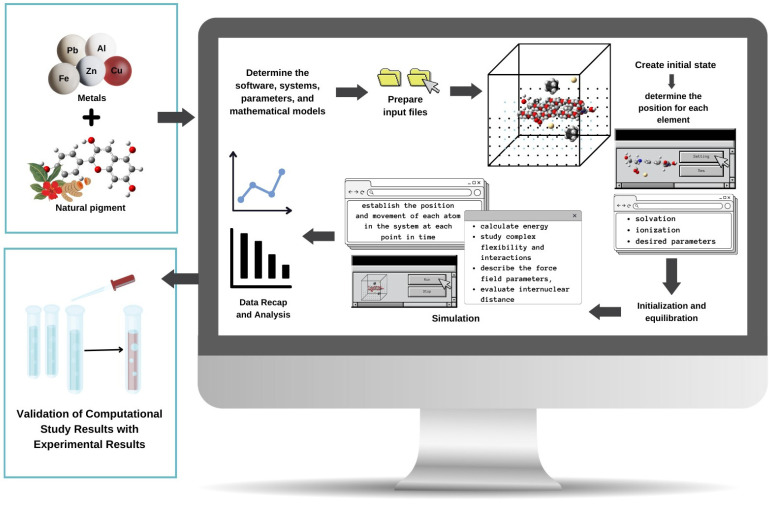
Illustration of Molecular Dynamics Simulation Flow to study the interaction between natural pigments and metals.

Molecular dynamics is a computational method that can be used in simulation to predict the position and movement of each atom in the system at each point in time based on a general model of physics [98,151]. In this simulation, the future speed and position of the atom are estimated based on its current speed and position by considering thermal motion [78]. As an illustration, the steps that can be carried out in molecular dynamics simulations to study interactions between natural pigments and metals and the influence of different parameters can be seen in Figure 3.

Recently, molecular dynamics has become increasingly popular because it is powerful and easy to access [152]. Its wide use is also related to its capabilities; it includes tests of various kinds of molecular behaviour as well as chemical systems as a function of time [98]. Molecular dynamics simulations can model important biomolecular processes, including conformational changes and conformation theory, binding of ligand molecules, and thermodynamic parameters, revealing the positions of all atoms at femtosecond temporal resolution. These simulations also allow researchers to describe the structure, thermodynamic properties, kinetic energy, surface potential energy, and dynamic properties of a system [78,98,152]. Table 7 presents the details of studies that have used molecular dynamics simulations to study metal–pigment interactions.

Moradi et al. (2021) [158] used molecular dynamics simulations to study the adsorption behaviour of the quercetin from *Satureja hortensis* L. extract (SHE) complexed with Zn on metal surfaces. SHE is derived from *S. hortensis* (synonym *Satureja postii* Arzn.), an aromatic plant from the Lamiaceae family [159]. The authors performed these simulations to understand the adsorption behaviour of Zn cation complexes with pigments on the surface of an Fe substrate. The authors performed modelling by using the Forcite module of the Material Studio software in a simulation box (2.73 × 2.73 × 3.31 nm). They attached a bottom Fe plate and an upper solvent layer, containing one complex and 800 water molecules, at a speed of 1500 ps in the NVT ensemble using a universal force field with a timeline of 1 fs and a temperature of 298 K. They observed interactions between the Zn(II)–quercetin complex and the substrate (Fe) in the water phase. The authors considered non-bonding interactions in the system to be an ‘atom-based simulation’ and electrostatic interactions adapted as an ‘ewald simulation’ approach. By balancing temperature and energy, the entire system under investigation reaches a state of equilibrium. The authors reported an absorption energy (E_ads_) of −42.4 kJ mol^−1^ for Zn(II)–quercetin, indicating that there is good adsorption strength between the adsorbate and the metal substrate. E_ads_ will greatly determine the strength of adsorption on the surface of the substrate: an increasingly negative value indicates a stronger interaction as well as stable and spontaneous absorption. The authors compared the theoretical results with the experimental results. The calculations could satisfactorily explain the experimental findings [160,161]. Based on the field emission scanning electron microscopy results, it is known that the addition of an inhibitor can coat Fe to prevent corrosion. Apart from that, the Fourier-transform infrared spectrum showed additional peaks that indicate the formation of Fe-O bonds from the complex and Zn-O bonds from the Zn hydroxide and its adsorption on metal surfaces. This is also in line with the UV–Vis spectroscopic results: a comparison of adsorption rates shows that the complex will be more adsorbed on the steel surface and form an inhibitory layer than dispersed in the electrolyte with and without the addition of inhibitors [158].

Moin and Hofer (2014) [155] conducted ab initio quantum mechanical charge field–molecular dynamics simulations using the HF method along with 6-31G** basis sets to study structural properties and dynamic properties and to observe the hydration behaviour of porphyrin and Mg–porphyrin complexes. Porphyrin is one of the most prevalent types of biochromes; this macrocyclic compound is the result of haem breakdown [139,162]. This simulation was done for Mg–porphyrin complexes with porphyrins inserted into a cubic box with a side length of 39.28 Å with B2000 explicit water molecules utilising periodic boundary conditions. The system was run in the NPT (isothermal–isobaric) ensemble using the Velocity–Verlet algorithm and at 298.15 K. The charge of the water molecules was adopted from the SPC/E water model. Based on the binding free energy and surface area exposed to solvents, these two confirmations have different hydration behaviours. Complexation of Mg ions with porphyrin will change the hydration pattern significantly, where the Mg–porphyrin complex has much better stability, as indicated by the stability of the penta-coordinated axial water molecules throughout the simulation. Compared with uncomplexed porphyrin, the Mg–porphyrin complex shows differences in molecular coordination that produce different H-bonding patterns. This is also supported by the vibrational power spectrum evaluated for both solutions obtained via the Fourier-transform velocity autocorrelation function observed in the quantum mechanical charge field–molecular dynamics simulation [155].

Singh et al., 2019 [156] also carried out molecular dynamics simulations with the AMBER14SB force field for standard groups and the GAFF force field for the remaining atoms in studying metallophilic interactions between closed-shell metal ions. In this research, a molecular dynamics study was carried out to evaluate the possibility of increasing or decreasing internuclear distance upon low-energy conformational changes in Ag in the middle of the porphyrin ring. To evaluate changes in the internuclear distance between Ag molecules, an analysis was carried out on the flexibility of the complex (Porphyrin-Ag) in vacuum for 100 ns. The root-mean-square deviation of the system was calculated during the simulation by using the minimised initial structure as a reference. The results obtained show that the trajectory attains relatively stable RMSD after approximately the first 20 ns, with an average value of 0.71 (0.36). In addition, the results in this study also show estimates of internuclear distances Ag···Ag, i.e., in the range 3.0–4.2 in the first cluster (average: 3.87(0.59)) and 3.9–4.9 in the second (average: 4.37(0.51)), where the results of molecular dynamics simulation are perfectly compatible with those predicted by EPR spectroscopy [156].

Huamán et al. (2021) [157] carried out molecular dynamics simulations of cyanidin-3-glucoside and bixin analysed in an isolated titanium dioxide (TiO_2_) dye/cluster system. Cyanidin is an anthocyanin that is responsible for the red and purple colours in berries, red sweet potatoes, and purple corn [163]. During calculations, the authors applied charge balance throughout the molecular system. They used the NVT ensemble at 300 K to maintain the temperature during the simulation under environmental conditions with mass conservation. The authors determined the molecular system’s energy by solving for potential and kinetic energy and for the binding energy of the dye–TiO_2_ complex. They subtracted the total energy of each component from the total energy of the complex. In addition, to study the dye–TiO_2_ electrode interaction, the authors employed complex molecular dynamics simulations using Reax FF. They found that chemisorption is carried out through a successive deprotonation process, and the formation of a bond between Ti-O and cyanidin-3-glucoside occurs by an OH retaining group and by a COOH retaining group for the bond between Ti-O and bixin, where bixin also shows greater affinity with the overly folded TiO_2_. Bixin also shows a tendency for dimer formation due to intermolecular π–π interactions that facilitate aggregation at the surface. The binding between Ti-O and cyanidin-3-glucoside is related to the knowledge that cyanidin-3-glucoside sensitisation is a monodentate anchor via the C4′ hydroxyl group of the benzene–diol molecule, where this interaction causes hydrogen bonding and passivation of TiO_2_ with protons from the glycosidic part of the molecule. The authors validated the calculations with UV–Vis spectroscopy and Fourier-transform infrared spectroscopy. They noted a broadening of the UV–Vis absorbance spectrum, evidence that electronic transfer occurs in the context of the formation of Ti-O bonds. Furthermore, Fourier-transform infrared spectroscopy showed that after sensitisation to TiO_2_, the peak associated with free OH bonds (3600 cm^−1^) disappear, forming a single band associated with hydrogen bonds, which includes inter- and intramolecular interactions, then C-H stretching from benzene becomes weaker and shifts to the red (2910 cm^−1^). Apart from that, one of the strongest pieces of evidence of an interaction is the presence of the Ti-O absorption band at 559 cm^−1^ [157,164].

Asadi et al. (2020) [120] carried out Monte Carlo/molecular dynamics simulations to study the interaction between the Zn–luteolin complex and Fe. Using the Adsorption Locator module in the Materials Studio software, the authors initially performed Monte Carlo simulation, using the Monte Carlo cells of the complexes as the initial configurations for the aqueous-phase molecular dynamics simulation. Next, the authors performed the molecular dynamics simulation in the NVT ensemble with a time step of 1 fs, a controlled temperature of 298 K, and in the COMPASS force field. The results showed that the Zn(luteolin)n (*n* = 2–5) complexes adsorb on the Fe surface. However, stability near the surface can only be achieved by the Zn(luteolin)2 and Zn(luteolin)3 complexes because of the tetrahedral-like geometry of large complexes. Subsequent investigation revealed that chemicals adsorb with parallel alignment to the surface. The authors calculated the adsorption energies: −691.78 kcal mol^−1^ for Zn(luteolin)2, −862.68 kcal mol^−1^ for Zn(luteolin)3, −1000.19 kcal mol^−1^ for Zn(luteolin) 4, and −1049.55 kcal mol^−1^ for Zn(luteolin)5. This negative value indicates that the adsorption that occurs is stable and spontaneous. The adsorption energy increases as the number of luteolin ligands bound to the Zn atom increases. The field emission scanning electron microscopy and atomic force microscopy results show that after mixing, there is a uniform film of a combination of Zn^2+^ cations and organic compounds of lemon balm extract in the specimen formed. Apart from that, grazing incidence X-ray diffraction showed that the formation of lower corrosion products (Fe oxide/hydroxide) occurs on the surface, a phenomenon that can be connected to the protective inhibitory film generation on the steel surface with the addition of an inhibitor, in this case a compound complexed with Zn [120].

Molecular dynamics simulations can be widely used to study metal–pigment interactions, including the simultaneous interactions between several metals and pigments. Moreover, various conditions can be simulated to evaluate their impact on the experimental results. Because molecular dynamics considers Newton’s laws of motion—where the spatial position of each atom as a function of time is predicted and then used to calculate the force of each atom—it can be used to predict how a molecular system will experience movement over time based on general models of physics [151,152]. The structure and properties of the system can also be described dynamically and thermodynamically. In simulations carried out in a solvent system, the solute and the simulated environment in the system can be controlled, and their position and movement can be known at each point in time; this is very difficult, or perhaps even impossible, to perform experimentally [165,166]. In addition, the components involved in the simulation are known in detail, such as the initial conformation of the compound used, the ligands that are bound, and what other molecules are present in the simulation systems. In this way, various impacts or influences from variations in conditions can also be identified in the simulation. Another advantage of molecular dynamics simulations is their ability to reveal the dynamic behaviour of water molecules and various ions in the system, and the accuracy of the modelled structure can also be checked and even refined. Based on Table 6, molecular dynamics simulations are consistent with experimental results. Therefore, the use of molecular dynamics simulations to study metal–pigment interactions is very useful and promising [152,167]. Although the application of molecular dynamics simulations is increasingly widespread, they have not been used to study heavy metal–natural pigment interactions. However, they have been used to analyse interactions between heavy metals and other components [168,169,170].

## 5. Conclusions

Quantum mechanics, including ab initio, DFT, and semiempirical methods, as well as molecular dynamics, have specific advantages and can be combined to complement each other. Among the available quantum mechanical methods, DFT with the B3LYP functional and the LANL2DZ ECP and basis set have been the most widely used due to their good accuracy and efficiency and relatively low cost. Molecular dynamics simulations allow one to study interactions in greater depth in various system conditions and to determine changes or predict dynamic movements over time. These simulations have been widely validated with various experimental studies. Considering their time, cost, and reliability, computational methods for preliminary studies and even for further studies of a system are very promising. Additional development and analysis of each method is still required, especially to determine the optimal combination of methods, approaches, functions, and basis sets to complement each other and increase accuracy and efficiency. Additional experiments are needed to find the best combination to study the bonds and interactions between heavy and non-heavy metals and natural pigments. Matters related to spin states and dispersion effects of metals on the development of metal indicators could also be a good part to evaluate in future research as the amount is very limited.

## Figures and Tables

**Table 1:** The maximum permissible limit of metals based on several regulatory agencies.

Elements	Limits (mg L^−1^)		
	FDA	WHO	EU
Heavy metals			
Antimony	0.006	0.02	0.005
Arsenic	0.01	0.01	0.01
Cadmium	0.005	0.003	0.005
Lead	0.005	0.01	0.01
Mercury	-	0.006	-
Non-heavy metals			
Chromium	0.10	0.05	0.05
Cobalt	-	0.05	-
Copper	1.00	2.00	2.00
Iron	0.30	2.00	0.20
Manganese	0.05	0.40	0.30
Nickel	0.10	0.02	0.02
Zinc	5.00	3.00	5.00

Note: EU, European Union; USFDA, United States Food and Drug Administration; WHO, World Health Organisation.

**Table 2:** Research related to the use of natural pigments as metal indicators.

Pigment	Metal	Color Change	Limit of Detection (Visually Visible)	Reference
Chlorophyll-based silver nanoparticle	Hg	Brown to light brown or colourless	60 µM	[58]
Curcumin-anthocyanin (hydrogel strips)	Cd	White to bluish green	0.2 µM	[59]
	Hg	White to blue	0.2 µM	
Curcumin (cellulose acetate sensor strip)	Pb	Yellow to orange	20 µM	[60]
Curcumin (cellulose nanofiber)	Pb	Orange to red	9 µM	[61]
Curcumin-gold nanoparticle	Hg	Reddish wine to light blue	2–10 µM	[62]
Curcumin (zein membrane)	Fe	Yellow to brown	7.16 µM	[56]
Cyanidin (solution)	Al	Purple to violet to blue	50 µM	[57]
	Cu	Violet to blue	50 µM	
	Fe	Pink to violet to blue	200 µM	
	Pb	Purple to violet to blue	80 µM	
Cyanidin (dipstick sensor)	Fe	White to pink	179–7162 µM	[63]

**Table 3:** Research on metal–natural pigment interactions have used ab initio methods.

Method	Approach	Purpose	Pigment	Metal	Compatibility with Experimental Results	Reference
Ab initio	HF method with the 6-31G(d) basis set	Optimise structures in various conformations	Flavone and flavylium	-	Compatible in terms of internal rotation barrier	[99]
Ab initio	HF method with the 6-31G basis set	Calculate molecular (hyper) polarisabilities and band structures	Porphyrin	Mg, Ni, Zn	-	[100]
Ab initio	HF method with the 6-31G* basis set	Calculate fully optimised structures and atomic charges	Bacteriochlorin	Mg	Compatible in terms of structures, slightly differences in terms of transition energy	[101]
IEFPCM-ab initio	IEFPCM at the HF/6-31G(d)//mPW1PW91/6-31G(d) level	Calculate solvation free energies	Flavylium	-	-	[102]
Ab initio	HF method with the 6-31G* basis set	Full geometry optimisation, study the influence of methylation position, and study the influence of additional functional groups in the induction of atomic charge distribution	Quercetin	Mg	Compatible in terms of binding free energy	[103]
			Luteolin	Mg		
Ab initio	HF method with the 6-31G basis set	Electronic characterisation	Anthracene, naphthalene, naphthacene, and pentacene	-	-	[104]

Note: HF, Hartree–Fock; QMCF-MD, quantum mechanical charge field–molecular dynamics; IEFPCM, Integral Equation Formalism for the Polarizable Continuum Model.

**Table 4:** Research on metal–natural pigment interactions that has used density functional theory methods.

Application	Approach	Condition	Purpose	Pigment	Metal	Complex	BE (kcal mol^−1^)	ΔBE (kcal mol^−1^)	ΔE (kcal mol^−1^)	ΔG (kcal mol^−1^)	ΔG _(binding)_ (kcal mol^−1^)	Molecular Energies (Hartree)	Ground State Energies (kcal mol^−1^)	Compatibility with Experimental Results	Reference
Gaussian 09	DFT/M052X/6-31+G(d) basis set (C, O, Al and H) and atoms - relativistic compact Stuttgart/Dres den effective core potential in conjunction with its split valence basis set for Cu and Fe atoms	Aqueous phase	Determine coordination properties	Quercetin	Al, Fe, and Cu	(4–5) Al(OH)H_4_Que+		13.7	0					Compatible	[17]
						(3′-4′) AlH_3_Que+		0.0	4.5						
						(3′-4′s-cis) Fe(OH)_2_H_3_Que		–	0.6						
						(4–5) CuH_4_Que+		28.2	0						
						(3′-4′H) CuH_4_Que+		53.4	4.7						
						(3–4s-cis) CuH_4_Que+		0.0	2						
Gaussian 03	DFT B3LYP/6-31+G(d)/LANL2DZ followed by single-point calculations using the 6-311+G(2d,2p) basis set	Gas phase	Locate the exact chelation site	Quercetin (3,30,40,5,7-pentahydroxylflavone)	Cr	Natural quercetin-bare Cr(III) ion	1238.703333							Compatible	[72]
		Ethanol phase				Natural quercetin-bare Cr(III) ion	668.98								
		Gas phase				Deprotonated quercetin-bare Cr(III) ion	1514.885								
		Ethanol phase				Deprotonated quercetin-bare Cr(III) ion	721.735								
		Gas phase				Deprotonated quercetin-hydrated Cr(III)	469.3525								
		Ethanol phase				Deprotonated quercetin-hydrated Cr(III)	78.4225								
Gaussian 16	DFT/M05–2X/6-311+G(d,p)		Study chelating properties	Apigenin	Cu and Fe	apigenin/H_3_A − Cu(II)				−2.2				-	[80]
						apigenin/H_2_A^−^ − Cu(II)				−4.6					
						apigenin/HA^2−^ − Cu(II)				−6.9					
						apigenin/H_3_A − Fe(III)				−7.0					
						apigenin/H_2_A^−^ − Fe(III)				−12.9					
						apigenin/HA^2−^ − Fe(III)				−18.2					
Gaussian 09	DFT/UB3LYP/6-31+G(d,p)/LANL2DZ	Gas phase	Geometry optimisation	Quercetin	Cu	i-quercetin − Cu(II)				−18.00 (kJ mol^−1^)				Compatible	[118]
		Solution phase								−41.03 (kJ mol^−1^)					
		Gas phase				ii-quercetin − Cu(II)				−9.31 (kJ mol^−1^)					
		Solution phase								−43.29 (kJ mol^−1^)					
		Gas phase				iii-quercetin − Cu(II)				334 (kJ mol^−1^)					
		Solution phase								−22.44 (kJ mol^−1^)					
		Gas phase		Chrysin		chrysin − Cu(II)				−8.31 (kJ mol^−1^)					
		Solution phase								−33.22 (kJ mol^−1^)					
Gaussian 03	DFT/B3LYP/6-31g**/CPCM	Methanol and water phase	Investigate interactions and geometry optimisation	Apigenin	Al	Al1B4,5Ap^2+^				−4.95				Compatible	[119]
						Al1B4,5Ap1^2+^				14.10					
				Luteolin		Al1B4,5Lu1^2+^				14.51					
DMol3 code	MD/DFT/GGA/PBE functional and DNP basis set	Solution phase	Investigate metal cation–pigment interactions	Luteolin	Zn	Zn(luteolin)2 (hydroxyl complexation)			2.585					Compatible	[120]
						Zn(luteolin)3 (hydroxyl complexation)			2.550						
						Zn(luteolin)4 (hydroxyl complexation)			2.526						
						Zn(luteolin)5 (hydroxyl complexation)			2.438						
						Zn(luteolin)2 (carbonyl complexation)			2.035						
						Zn(luteolin)3 (carbonyl complexation)			2.093						
						Zn(luteolin)4 (carbonyl complexation)			2.170						
						Zn(luteolin)5 (carbonyl complexation)			2.131						
Gaussian 16	DFT/B3LYP/6-31+G(d,p)/LANL2DZ		Structure optimisation	Naphthoquinone	Pd, Ni, and Co	[Co(L1)_2_(H_2_O)_2_].2H_2_O			0.29 (eV)	−581.21 kJ mol^−1^				-	[121]
						[NiL1(H_2_O)_2_(CH_3_COO^−^)]			0.18 (eV)	−631.88 kJ mol^−1^					
						[PdL1 (H_2_O)Cl]			0.39 (eV)	−501.43 kJ mol^−1^					
						[CoL2(H_2_O)_2_(CH3COO^−^)]			0.32 (eV)	−434.27 kJ mol^−1^					
						[NiL2(H_2_O)_2_ (CH_3_COO^−^)].H_2_O			0.71 (eV)	−414.27 kJ mol^−1^					
						[PdL2 (H_2_O)Cl]			0.34 (eV)	−301.43 kJ mol^−1^					
Gaussian 09	DFT/B3LYP/6-31++G(d,p)/PCM	Aqueous phase	Structure optimisation	Cyanin	Na+			−12.21			1.16			Compatible	[82]
					K^+^			−6.36			10.58				
					Mg(II)			−25.26			−13.50				
					Ca(II)			−17.62			−2.71				
					Cr(II)			−42.46			−28.22				
					Mn(II)			−30.69			−17.85				
					Fe(II)			−32.40			−19.75				
					Co(II)			−31.26			−19.56				
					Ni(II)			−32.40			−20.02				
					Cu(II)			−52.04			−37.59				
					Zn(II)			-31.74			−19.65				
					Al(III)			−63.42			−53.97				
					Cr(III)			−72.62			−63.54				
					Fe(III)			−80.64			−73.44				
					Co(III)			−108.66			−100.94				
Gaussian 98	DFT/B3LYP/6-31G* basis set for C and H atoms and 6-31+G* basis set for O atoms/LanL2DZ/PCM continuum model	Solution with ε = 78.4 (corresponding to bulk water)	Structure optimisation	Quercetin	Cu	OQ1Cu				−302.7				Compatible	[122]
						OQ2Cu				−336.1					
						OQ3Cu				−308.2					
						SQ1Cu				−322.8 (−312.5)					
						SQ2Cu				−323.3 (−328.8)					
						SQ3Cu				−302.7 (−301.5)					
						D1Cu				−500.2					
						D2Cu				−499.8					
						DD3Cu				−676.7					
Gaussian 03	DFT/M052 × 29/6-31+G(d) b/PCM	Ethanol (ε = 24.85)	For full optimisation	Quercetin	Al	a3-4eq H4QueAl(H_2_O)_2_(OH)_2_			2.9					Compatible	[73]
						a3-4ax H4QueAl(H_2_O)_2_(OH)_2_			3.6						
						a4-5eq H4QueAl(H_2_O)_2_(OH)_2_			1.2						
						a4-5ax H4QueAl(H_2_O)_2_(OH)_2_			0						
						b3-4eq H3QueAl(H_2_O)_3_(OH)			5.1						
						b3-4ax H3QueAl(H_2_O)_3_(OH)			7.4						
						b4-5eq H3QueAl(H_2_O)_3_(OH)			5.9						
						b4-5ax H3QueAl(H_2_O)_3_(OH)			7.4						
Gaussian 03	DFT/B3LYP/6-31+G (d)/LANL2DZ followed by a single-point calculation using a different basis set (6-311++G(d,p))	Water phase	Geometry optimisation	Chalcone (butein)	Mg, Cr, Fe, and Cu	Cu^2+^–O2′O9	127							-	[117]
						Cu^2+^–O4O3	125								
						Fe^2+^–O2′O9	32								
						Fe^2+^–O4O3	21								
						Mg^2+^–O2′O9	30								
						Mg^2+^–O4O3	22								
						Cr^2+^–O2′O9	46								
						Cr^2+^–O4O3	26								
		Gas phase		Chalcone (butein)	Mg, Cr, Fe, and Cu	Cu^2+^–O2′O9	517		0.03187 (Hertee)						
						Cu^2+^–O4O3	493								
						Fe^2+^–O2′O9	461		0.0716 (Hertee)						
						Fe^2+^–O4O3	406								
						Mg^2+^–O2′O9	423		0.06548 (Hertee)						
						Mg^2+^–O4O3	370								
						Cr^2+^–O2′O9	462		0.09428 (Hertee)						
						Cr^2+^–O4O3	397								
Gaussian09W and GaussView 6.0.16	DFT/6-31+G(d)/B3LYP		Investigate stability, reactivity, nature of interaction, and application of the complexes	Quercetin (5-hydroxy-4-keto group)	Al				2.8297 (eV)					Compatible	[65]
					Mg				1.2679 (eV)						
					Na				3.1149 (eV)						
					K				3.2172 (eV)						
					Ca				0.6134 (eV)						
					Al				2.603 (eV)						
					Mg				1.3749 (eV)						
					Na				3.3362 (eV)						
					K				3.0782 (eV)						
					Ca				1.3983 (eV)						
				Quercetin (O3′/O4′ ortho-dihydroxyl (catechol) group)	Al				2.8828 (eV)						
					Mg				2.3431 (eV)						
					Na				2.8488 (eV)						
					K				3.1038 (eV)						
					Ca				1.156 (eV)						
					Al				1.051 (eV)						
					Mg				1.076 (eV)						
					Na				1.739 (eV)						
					K				1.697 (eV)						
					Ca				0.854 (eV)						
Gaussian 16	DFT/M06-2X/def2-SVP	Gas phase	Investigate electronic and structural properties of morin	Quercetin	Fe and Cu	Cu(II)M2 6 m							−2,411,782	Compatible	[123]
						Fe(III)M2 6 m							−2,175,328		
						Cu(II)Q2 6 m							−2,411,784		
						Fe(III)Q2 6 m							−2,175,331		
						Cu(II)M2 5 m							−2,411,780		
						Fe(III)M2 5 m							−2,175,325		
						Cu(II)Q2 5 m							−2,411,769		
						Fe(III)Q2 5 m							−2,175,326		
Gaussian 09	DFT/B3LYP/6-31+G-(d, p)		Structural analysis	Cyanidin	Zn				1.47 (eV)					-	[124]
Gaussian 03	DFT/B3LYP/6-31G*/LANL2DZ followed by single-point calculations using the extended 6-311++G** basis set	Gas phase	Geometry optimisation	Quercetin	Fe	I-Q-Fe^2+^			12.3					Compatible	[76]
						II-Q-Fe^2+^			10.1						
						III-Q-Fe^2+^			0						
						I-Q^−^-Fe^2+^			27.4						
						II-Q^−^-Fe^2+^			23.6						
						III-Q^−^-Fe^2+^			5.6						
						IV-Q^−^-Fe^2+^			0						
						V-Q^−^-Fe^2+^			34.2						
						VI-Q^−^-Fe^2+^			35.3						
						VII-Q^−^-Fe^2+^			48.3						
						VIII-Q^−^-Fe^2+^			41.7						
						I-Q^−^-Fe^2+^(H_2_O)_4_			13.8						
						II-Q^−^-Fe^2+^(H_2_O)_4_			12.9						
						III-Q^−^-Fe^2+^(H_2_O)_4_			0						
						IV-Q^−^-Fe^2+^(H_2_O)_4_			1.8						
						V-Q^−^-Fe^2+^(H_2_O)_4_			41						
						VI-Q^−^-Fe^2+^(H_2_O)_4_			50.5						
						VII-Q^−^-Fe^2+^(H_2_O)_4_			50.5						
						VIII-Q^−^-Fe^2+^(H_2_O)_4_			42.3						
						I-2Q^−^-Fe^2+^			0						
						II-2Q^−^-Fe^2+^			5.9						
						III-2Q^−^-Fe^2+^			1.6						
						IV-2Q^−^-Fe^2+^			2.5						
						I-2Q^−^-Fe^2+^(H_2_O)_2_			12.9						
						II-2Q^−^-Fe^2+^(H_2_O)_2_			5.2						
						III-2Q^−^-Fe^2+^(H_2_O)_2_			0						
						IV-2Q^−^-Fe^2+^(H_2_O)_2_			9.3						
Gaussian 16	DFT/M05-2X/6-31+G(d) (C, H, O, and Al) and relativistic compact Stuttgart/ Dresden effective core potential with its related split valence (Cu and Fe)	Aqueous phase	Properties and geometry optimisation	Luteolin	Al, Fe, and Cu	Al(OH)2(H2O)2(H3Lu) (3′-4′)			4.9	−89.9				-	[64]
						Al(OH)2(H2O)2(H3Lu) (4–5)			0	−94.8					
						Fe(OH)2(H2O)2(H3Lu) (3′-4′)			1.4	−82.4					
						Fe(OH)2(H2O)2(H3Lu) (4–5)			0	−83.9					
						[Fe(H2O)3(OH)(H3Lu)](3′-4′)			2.8	−61.7					
						[Fe(H2O)3(OH)(H3Lu)](4–5)			0	−64.4					
						Cu(OH)2(H2O)2(H3Lu)(3′-4′)			0.2	−61.1					
						Cu(OH)2(H2O)2(H3Lu)(4–5)			0	−61.2					
Gaussian 09	DFT/M052X/6-31+G(d)	Water phase	Studied the complexation	Curcumin	Al and Fe	[Al(H2O)3(OH)(LA)]+				−135.1				Compatible	[125]
						[Al(H2O)3(OH)(LB)]				−124.9					
						[Fe(H2O)(OH)3(LA)]				−57.1					
						[Fe(H2O)(OH)3(LB)]				−55.5					
Gaussian 09	DFT/U-B3LYP/6-31G* and relativistic effective core potential with a valence basis set/LANL2DZ		Coordination and geometry optimisation	Quercetin	Ni	[Ni(L1)(fla)]ClO4			3.056 (eV)					Compatible	[71]
						[Ni(L1)(fla)]ClO4			3.045 (eV)						
						[Ni(L3)(fla)]ClO4			3.029 (eV)						
						[Ni(ntb)(fla)]+			3.033 (eV)						
Gaussian 09	DFT/UB3LYP*/6-311++G(d,p)		Full geometry optimisation	Phenoxazines	Co	I(R = R_1_ = CH_3_)_LS_Co^III^-SQ			4.5					-	[70]
						I(R = R_1_ = CH_3_)_HS_Co^III^-Q									
						I(R = CH_3_, R1 = CF_3_)_LS_Co^III^-SQ			−1.7						
						I(R = CH_3_, R1 = CF_3_)_HS_Co^III^-Q									
						I(R = R_1_ = CF_3_)_LS_Co^III^-SQ			−8.7						
						I(R = R_1_ = CF_3_)_HS_Co^III^-Q									
						II(R_2_ = H)_LS_Co^III^-SQ			14.4						
						II(R_2_ = H)_HS_Co^III^-Q									
						II(R_2_ = CH_3_)_LS_Co^III^-SQ			12.1						
						II(R_2_ = CH_3_)_HS_Co^III^-Q									
						II(R_2_ = Ph)_LS_Co^III^-SQ			3.4						
						II(R_2_ = Ph)_HS_Co^III^-Q									
						III(R_2_ = H)_LS_Co^III^-SQ			14.4						
						III(R_2_ = H)_HS_Co^III^-Q									
						III(R_2_ = CH_3_)_LS_Co^III^-SQ			11.9						
						III(R_2_ = CH_3_)_HS_Co^III^-Q									
						III(R_2_ = Ph)_LS_Co^III^-SQ			3.3						
						III(R_2_ = Ph)_HS_Co^III^-Q									

Note: BE, energy associated with the formation of bonds between two or more molecules in a system; ΔBE, change in energy associated with the formation of bonds between two or more molecules in a system; ΔE, change in molecular system energy; ΔG, change in Gibbs free energy of the molecular system; ΔG (binding), change in Gibbs free energy associated with the formation of a bond between two or more molecules.

**Table 5:** Research on heavy metal–natural pigment interactions that has used density functional theory methods.

Application	Approach	Condition	Purpose	Pigment	Metal	Complex	EE (eV)	ΔE (eV)	ΔE_DIS_ (kJ mol^−1^)	Molecular Energy (Hartree)	Compatibility with Experimental Results	Reference
Gaussian 09	DFT/B3LYP/6- 31G/LANL2DZ		Determine the reactivity and stability of the complex	Porphyrin	Pb, Cd, Hg, Sn, and As	Pb-TMPyP				−2138.3	Compatible	[139]
						Cd-TMPyP				−2182.9		
						Hg-TMPyP				−2177.5		
						Sn-TMPyP				−2137.1		
						As3+-TMPyP				−2140.4		
						As5+-TMPyP				−2138.9		
						Pb-TDMImP				−2207.3		
						Cd-TDMImP				−2251.9		
						Hg-TDMImP				−246.5		
						Sn-TDMImP				−2205.9		
						As3+-TDMImP				−2209.3		
						As5+-TDMImP				−2207.8		
						Pb-TDMPzP				−2207.2		
						Cd-TDMPzP				−2251.8		
						Hg-TDMPzP				−2246.4		
						Sn-TDMPzP				−2206.0		
						As3+-TDMPzP				−2209.3		
						As5+-TDMPzP				−2207.9		
Gaussian 03	DFT/B3LYP/6-31G(d,p)/LANL2DZ	Methanol phase	Geometry optimisation	Quercetin	Pb	Pb(II)-quercetin		0.124			Compatible	[135]
Gaussian 09	DFT/B3LYP/LANL2DZ followed by TD-DFT/LC-wPBE	Gas phase	Geometry optimisation and energy calculations	Astaxanthin	Pb, Cd, and Hg	[ASTA-Pb]^+2^	2.05				Compatible	[128]
						[ASTA-Pb_2_]^+4^	1.84					
						[ASTA-Cd(H_2_O)_2_]^+2^	1.85					
						[ASTA-Cd_2_(H_2_O)_4_]^+4^	2.08					
						[ASTA-Hg(H_2_O)_2_]^+2^	1.93					
						[ASTA-Hg_2_(H_2_O)_4_]^+4^	2.06					
		Ethanol phase				[ASTA-Pb]^+2^	1.96					
						[ASTA-Pb_2_]^+4^	1.82					
						[ASTA-Cd(H_2_O)_2_]^+2^	1.68					
						[ASTA-Cd_2_(H_2_O)_4_]^+4^	2.41					
						[ASTA-Hg(H_2_O)_2_]^+2^	1.82					
						[ASTA-Hg_2_(H_2_O)_4_]^+4^	2.40					
Gaussian 03	DFT/B3LYP/6-311G(d,p)/LANL2DZ		Calculation of complete optimisation	Pterins, isoxanthopterin, sepiapterin	Cd, Hg	Pterins-Cd			7.5		Compatible	[140]
						Pterins-Cd+1			270.3			
						Pterins-Cd+2			762.7			
						Pterins Hg			4.6			
						Pterins-Hg+1			252.7			
						Pterins-Hg+2			797.1			
						Isoxanthopterin-Cd			7.5			
						Isoxanthopterin-Cd+1			276.6			
						Isoxanthopterin-Cd+2			778.2			
						Isoxanthopterin-Hg			5.9			
						Isoxanthopterin-Hg+1			258.6			
						Isoxanthopterin-Hg+2			807.5			
						Sepiapterin-Cd			12.9			
						Sepiapterin-Cd+1			366.9			
						Sepiapterin-Cd+2			1018.4			
						Sepiapterin-Hg			9.2			
						Sepiapterin-Hg+1			346.4			
						Sepiapterin-Hg+2			1032.2			

EE, energy related to the excitation process or energy given to a system related to changes in state from the ground state to the excited state; ΔE, change in molecular system energy; ΔE_DIS_, change in energy during the dissociation process.

**Table 6:** Research on metal–natural pigment interactions that has used semiempirical methods.

Method	Approach	Purpose	Pigment	Metal	Compatibility with Experimental Results	Reference
Semiempirical method	AM1 using HyperChem program	Study complexation processes	Anthocyanin	Al	Compatible	[142]
Semiempirical method	MM^+^ and AM1	Molecular calculation	Anthocyanin	Al and Ga	Compatible	[143]
Semiempirical method	AM1 Hamiltonian	Calculate the structural modifications caused by the chelation process	Quercetin	Al	Compatible	[74]
Semiempirical method	ZINDO/S CIS (40,40) or (45, 45) levels	Calculate transition energies and oscillation strengths, estimate corresponding spectroscopic transition energy values, and study the existence of dark electronic states in the system	Bacteriochlorin	Mg	Compatible	[106]
Semiempirical method	PM5	Study orbitals	Bacteriochlorin, chlorin, and porphin	Mg	Compatible	[141]
Semiempirical method	AM1 using MOPAC17 version 6	Geometry optimisation and electronic structure analysis	Xanthophylls, antheraxanthin, lutein, neoxanthin, violaxanthin, and zeaxanthin	-	Compatible	[144]
Semiempirical method	PM5 using the MOPAC 2002 package	Structure optimisation	Chlorophyll and chlorophyll d peptides	Mg	Compatible	[145]
Semiempirical method	AM1 (available in the AMPAC package)	Structure optimisation	Malvidin	-	Compatible	[146]
Semiempirical molecular dynamics	PM6 with Grimme D3 dispersion correction	Investigate intermolecular behaviour	Anthocyanins (malvidin-3-glucoside)	Al and Sn	Compatible	[147]
ONIOM semiempirical method	PM6 and Pm3MM	Solvation optimisation	Anthocyanins	Al, Ga, Cr, Fe, and Mg	-	[138]
QM/MM semiempirical method	Pm3MM	Solvation optimisation	Anthocyanins	Mg, Al, Ga, Sn, Cr, and Fe	Compatible	[78]

Notes: ONIOM, Our Own N-layered Integrated molecular Orbital and molecular Mechanics; QM/MM, Quantum Mechanics/Molecular Mechanics.

**Table 7:** Research on metal–natural pigment interactions that has used molecular dynamics simulation.

Method	Approach	Purpose	Pigment	Metal	Compatibility with Experimental Results	Reference
Molecular dynamics simulations	-	Calculate energy-minimised structures	Hesperidin, rutin, neodiosmin, diosmin, and neohesperidin	Co	Compatible	[153]
Molecular dynamics simulations	AMBER force field (GAFF) and TIP3P model using the Sander module in the Amber 10.0 simulation package	Identify several conformations of the co-pigmentation complex	Oenin	-	Compatible	[154]
Molecular dynamics simulations	restrained electrostatic potential (RESP) protocol implemented in the ANTECHAMBER module of AMBER 11	Partial atomic charges determination	Quercetin and luteolin	Mg	Compatible	[108]
	AMBER force field (GAFF)	Describe the force field parameters of the substrates				
	AMBER ff99SB force field	Potential determination				
Ab initio QMCF-MD simulations	HF method with the 6-31G** basis set; Amber force field (GAFF) and restrained electrostatic potential (RESP)	Study structural and dynamic properties, and observe hydration behaviour	Porphyrin	Mg	Compatible	[155]
Molecular dynamics simulations	AMBER14SB force field and GAFF force field	Evaluate the possibility of increasing or decreasing internuclear distance upon low-energy conformational changes and assessing the flexibility of the complex	Porphyrin	Ag	Compatible	[156]
Monte Carlo/ molecular dynamics simulations	Materials Studio in the NVT ensemble with the COMPASS force field	Analyse the interaction of complexes	Luteolin–Zn complex	Fe	Compatible	[120]
Monte Carlo/ Molecular dynamics simulations	AMBER	Study conformation theories, thermodynamic parameters and movement rules of the molecular machine and kinetic energy to the potential energy surface	Cyanidin, delphinidin, petunidin	Mg, Al, Ga, Sn, Cr, and Fe	Compatible	[78]
Molecular dynamics simulations	Reax FF force field using NVT ensemble	Analyse interactions between components	Cyanidin-3-glucoside	Ti	Compatible	[157]
Molecular dynamics simulations	Forcite module of a Material Studio software in NVT ensemble employing universal forcefield	Study adsorption behaviour	*Satureja hortensis* extract (isoferulic acid, caffeic acid, kuersetin, rosmarinic acid, apigenin glucoside, and chlorogenic acid)	Zn	Compatible	[158]

## Data Availability

Data sharing not applicable.
